# The changes of morphological and physiological characteristics in hemiparasitic *Monochasma savatieri* before and after attachment to the host plant

**DOI:** 10.7717/peerj.9780

**Published:** 2020-08-19

**Authors:** Lanlan Chen, Zaibiao Zhu, Qiaosheng Guo, Jun Guo, Zhigang Huang, Hui Zhang

**Affiliations:** 1 Institute of Chinese Medicinal Materials, Nanjing Agricultural University, Nanjing, China; 2 Huizhou Jiuhui Pharmaceutical Co., Ltd., Huizhou, China

**Keywords:** Anatomical structure, Artificial cultivation, Haustorium, Physiological performance, Root hemiparasite

## Abstract

**Background:**

*Monochasma savatieri* is an endangered hemiparasitic medicinal plant with a variety of antioxidant, antimicrobial and anti-inflammatory properties. Despite the urgent need to understand the parasitic biology of *M. savatieri*, parasite-host associations have long been neglected in studies of *M. savatieri*.

**Methods:**

We conducted a pot cultivation experiment to analyze changes in the growth traits, physiological performance and anatomical structures of *M. savatieri* grown with the potential host *Gardenia jasminoides* E., before and after the establishment of the parasite-host association.

**Results:**

Prior to the establishment of the parasite-host association, the presence of the host had no significant effect on the maximum root length, leaf indexes or total dry weight of *M. savatieri* seedlings, but had significant positive effect on seedling height, number of roots or number of haustoria. When it was continuously grown without a host, *M. savatieri* growth was rather slow. The establishment of the parasite-host association enhanced the growth of *M. savatieri*, and higher levels of photosynthetic pigments, increased antioxidant enzyme activity and lower malondialdehyde accumulation were observed in *M. savatieri* with an established parasite-host association. Furthermore, an analysis of the anatomical structures of *M. savatieri* showed that the establishment of the parasite-host association enabled better development of the seedling vegetative organs than that in seedlings without parasite-host associations.

**Conclusions:**

Our study demonstrates the physiological and anatomical changes that occurred in *M. savatieri* after connection with a host and suggests that the enhanced growth and development of *M. savatieri* were highly dependent on the parasite-host association.

## Introduction

*Monochasma savatieri* Franch. ex Maxim (Orobanchaceae) is a perennial root hemiparasite that grows on sunny hills in sparse forests and is only distributed in Southeast China and Kyushu (Amakusa Islands), Japan (Hong et al., 1998; Yamazaki, 1993; Zhang et al., 2015). *M. savatieri* parasitizes at least 27 host species from 25 families, which are mostly shrubs and herbs (Zhang et al., 2015). The whole dried plant of *M. savatieri* is widely applied as an herbal medicine and has antioxidant, antimicrobial and anti-inflammatory properties (Liu et al., 2013; Shi et al., 2013). *M. savatieri* is also a necessary component of proprietary Chinese medicines such as Yanning sirup, which has been used for the treatment of upper respiratory tract infections, tonsillitis and urinary tract infections (Committee of National Pharmacopoeia, Pharmacopoeia of P.R. China, 2015). Due to its unsustainable exploitation as well as habitat destruction, wild *M. savatieri* populations have decreased sharply in the wild (Zhang et al., 2015). In Japan, *M. savatieri* is also an endangered and threatened species (Environment Agency of Japan, 2000). To date, there are two reports on using tissue culture techniques to regenerate *M. savatieri*; one report was not aware of its hemiparasitism, and the other report did not focus on its association with a host (Yang, 2009; Zhang et al., 2017). Although a study on *M. savatieri* growth from seeds has been reported, the plants were cultivated without a host (Chen et al., 2020). The parasitic habit of *M. savatieri* has been known since 2015, and the basic biology of *M. savatieri* has scarcely been documented (Zhang et al., 2015). Given its medicinal values and industrial importance, there is a clear need to have a better knowledge of the parasitism of *M. savatieri*.

Root hemiparasitic plants can produce carbohydrates by photosynthesis but partially rely on their attachment to host roots for water, nutrients and carbon (Těšitel et al., 2011). The degrees of parasitism vary in root hemiparasitic plants (Irving & Cameron, 2009). Some depend on a host to survive (obligate parasites), whereas others can grow without a host (but typically grow better when attached to a host; facultative parasites). The life cycles of obligate parasites and facultative parasites in Orobanchaceae include two key developmental phases, the independent phase and the compatible phase. In the independent phase, the seedlings of obligate parasites develop independently for a short period of time until the stage when they attach to a host, whereas the seedlings of facultative parasites establish autotrophically before they attach to a host (Joel, 2013). The development of obligate parasites is coordinated with that of the host in the compatible phase. However, the compatible phase of facultative parasites refers to the period after attachment to a host (Joel, 2013). In all circumstances, haustoria attachment between the roots of the parasite and host is essential for successful establishment of parasite-host association.

Once the haustorium attaches to the surface of the host root, the rupture and penetration of the host tissues begin. A crucial step for the parasites in establishing parasite-host associations is the invasion of host tissues because natural resistance exists in some hosts (Pérez-de-Luque, 2013). After the attachment of the parasite to a host, the established functional haustorium extracts water, nutrients and carbon from its connected hosts (Těšitel et al., 2011). Additionally, studies of *Cuscuta* have shown that the functional haustorium transfers macromolecules such as mRNAs, proteins, secondary metabolites and small RNAs, as well as pathogens such as phytoplasma and viruses (Kim & Westwood, 2015; Shahid et al., 2018). Although growth stimulation after attachment to a host has been observed in several facultative parasites (Klaren & Janssen, 1978; Sui et al., 2019), changes in parasites before and after attachment to a host have rarely been experimentally tested (Li et al., 2012). The microscopic structures of haustoria have been the focus of investigation in root hemiparasites (Zhang et al., 2012), whereas investigations of microscopic structures of leaves and stems have received comparatively little attention. The observed morphological changes are significant in mature *M. savatieri* (Zhang et al., 2015), and *M. savatieri* is capable of autotrophic growth but remains small without a host (Chen et al., 2020); therefore, it is relevant to study the physiological and structural changes during the establishment of the parasite-host associations.

In this experiment, we study the changes in the growth traits, physiological performance and anatomical structures of *M. savatieri*. *M. savatieri* was grown with one host species and harvested at two developmental phases. The following questions were raised: (1) Does the presence of a host have an effect on growth responses or morphological and physiological changes in *M. savatieri* prior to the establishment of the parasite-host association? (2) How strong is the effect of the establishment of the parasite-host association on *M. savatieri*? The obtained results will not only help further our understanding of the parasitic biology of *M. savatieri* but will also lay a scientific foundation for the establishment of artificial cultivation systems for *M. savatieri* or similar underinvestigated root hemiparasites.

## Materials and Methods

### Plant materials and growth conditions

Fully ripe seed capsules of *M. savatieri* were collected from Yongfeng County, Jiangxi Province, China, in May 2017 and then exposed to air indoors for five days, after which they were stored in envelopes at 4 °C until they were needed. *Gardenia jasminoides* E. (Rubiaceae) was used as the host because it is commonly found in natural habitats of *M. savatieri* (Zhang et al., 2015) and was a pot host for *M. savatieri* under artificial cultivation conditions (L. Chen and Z. Zhu, 2017–2018, personal observations). On 19 March 2018, 60 7-month-old *G. jasminoides* seedlings of the same height (approximately 15 cm) obtained from a market were washed thoroughly in tap water and then transplanted into the center of a polyethylene plastic pot containing 770 g of a mix of nutritive soil and fine sand (1:2, v/v). One *G. jasminoides* seedling was planted in each pot. The pot was 10 cm in height, 15 cm in upper diameter and 7 cm in lower diameter. The cultivation substrate had 13.51 g·kg^−1^ soil organic matter, 0.57 g·kg^−1^ total *P*, 12.49 g·kg^−1^ total *K*, 70.95 mg·kg^−1^ available *N*, 7.65 mg·kg^−1^ available *P* and 173.45 mg·kg^−1^ available *K*. The pH of the substrate was 5.5. These soil properties were determined following the routine procedures for soil analysis described by Lu (2000) at the beginning of the experiment. One month after planting the host, *M. savatieri* seeds were removed from their seed capsules and pretreated with an 800 mg·L^−1^ gibberellin solution for 24 h at room temperature to promote seed germination and then rinsed thoroughly with sterile deionized water (Chen et al., 2018). The seeds were divided into two groups. One group included *M. savatieri* growing with *G. jasminoides* (+GJ), and the other group included *M. savatieri* growing without *G. jasminoides* (−GJ). Each group had 60 pots, with a total of 120 pots for the experiment. The seeds of *M. savatieri* are tiny, and the 1,000-seed weight is 0.07 g. To evenly disperse the *M. savatieri* seeds, 50 seeds per pot were mixed with 2 mL fine sand, sown on 17 April 2018 and maintained on the surface of the substrate mixture. At 4 weeks after sowing (day 28), at the two-leaf stage, vigorously growing seedlings of approximately 5 mm height were thinned to 10 per pot. Two *M. savatieri* seedlings were kept at a distance of approximately 2 cm apart from each other to reduce distance effects in each pot.

All plants were cultivated under natural day/night conditions in the greenhouse of the Institute of Chinese Medicinal Materials at Nanjing Agricultural University. The experiment was conducted from mid-March to early August in 2018, with daily temperatures ranging from 16 °C to 40 °C. To create light conditions close to those of the natural habitat of *M. savatieri*, after the *M. savatieri* seedlings were thinned out, a sunblock shade net that blocked approximately 50% of incident sunlight was placed on the greenhouse between May and August. During the experiment, all the pots were watered daily to field capacity with tap water. The pots were placed completely at random and were re-randomized every two weeks to reduce position effects.

### Harvest and sampling

*Monochasma savatieri* plants in the two groups were harvested at two key stages of parasite development (Joel, 2013): (1) 8 weeks after sowing (early seedling stage, prior to the establishment of the parasite-host association, PE) and (2) 16 weeks after sowing (late seedling stage, after the establishment of the parasite-host association, AE). The successful connection between *M. savatieri* and *G. jasminoides* was confirmed by the enhanced growth of *M. savatieri* (Klaren & Janssen, 1978; Sui et al., 2019). At each sampling point, 30 pots with *G. jasminoides* and 30 pots without *G. jasminoides* were randomly selected. Under each treatment, nine replicate pots were harvested for plant growth indicators and haustorium formation analyses (one *M. savatieri* per pot was randomly harvested); six replicates were taken to determine biomass (each replicate included seven seedlings from four replicate pots); three replicates were taken to determine plant physiological parameters (each replicate included a weighted sample from ten replicate pots). In addition, leaves, stem segments below leaves and lateral roots of *M. savatieri* were preserved in FAA (formaldehyde: glacial acetic acid: 50% ethanol; 1:1:18) for further examination of their anatomical structures. At each sampling, the leaves and lateral roots of *M. savatieri* were prefixed in 2.5% glutaraldehyde (Solarbio, Beijing, China) for 2 h at room temperature and then stored at 4 °C for SEM.

### Determination of plant growth

At harvest, whole *M. savatieri* plants were carefully dug up and then washed thoroughly in running water to remove soil and debris. A haustorium was carefully disconnected from the host roots under a stereomicroscope (Leica M165FC; Leica Microsystems, Germany) and pooled with the *M. savatieri* plants. The plants were placed in round Petri dishes containing distilled water, and the seedling height (SH), maximum root length (RL), number of leaves (L) and the number of root (R) were determined. Leaf morphological indexes, including leaf length (LL), leaf width (LW) and leaf area (LA), were measured using ImageJ (version 1.48; National Institutes of Health, Bethesda, MD, USA). As the seedlings of *M. savatieri* were weak prior to the establishment of the parasite-host association, the total dry weight (DW) of seven randomly selected seedlings was determined after oven-drying at 80 °C to a constant weight.

To facilitate the analysis of the haustoria, the roots stored in FAA were washed with deionized water, cleared in 10% KOH, stained in a 1% Safranin O solution, and soaked in a clearing solution (2.5 g·mL^−1^ chloral hydrate and 50% (v/v) glycerol) for 1 h as described previously with minor modifications (Cui et al., 2016). The number of haustoria (H) and presumably functional haustoria (PFH, haustoria with a xylem bridge) formed by each *M. savatieri* plant were counted with a bright-field microscope (Leica DM6B; Leica Microsystems, Germany).

### Assay of photosynthetic pigments

Fresh *M. savatieri* leaves of approximately 80 mg were cut into pieces and placed in a test tube containing 8 mL of 95% (v/v) ethanol in the dark at room temperature for 48 h. The contents of chlorophyll a (chl a), chlorophyll b (chl b) and carotenoids (car) were determined by measuring the absorbance of the supernatant at 665, 649 and 470 nm with a UV-1800 spectrophotometer (MaCy, China) (Lichtenthaler, 1987).

### Assays of total soluble sugar, soluble protein, proline and cell membrane permeability

The content of total soluble sugar (TSS) was assayed by the anthrone method and determined at 620 nm by a UV-1800 spectrophotometer (Fales, 1951). The amount of soluble protein (SP) was determined using the Coomassie brilliant blue assay, and its absorbance at 595 nm was measured by a UV-1800 spectrophotometer (Bradford, 1976). The proline was extracted by 15% (w/v) sulfosalicylic acid, and the supernatant was mixed with acetic acid and acid ninhydrin. The mixture was separated in toluene and the upper layer was used to determine the proline content by a UV-1800 spectrophotometer at 520 nm (Bates, Waldren & Teare, 1973). Fresh *M. savatieri* leaves were rinsed thoroughly with deionized water and then incubated in 30 mL deionized water at 25 °C, followed by shaking for 30 min. Electrolyte leakage was measured with a conductivity meter (EC400; Extech, Waltham, MA, USA). The solution was heated to 100 °C for 30 min and then cooled to room temperature. The total electrolytes in the solution were measured again and then expressed using the relative electrolyte leakage rate (Yang et al., 2012).

### Assays of malondialdehyde and antioxidant enzyme activities

The amount of malondialdehyde (MDA) was determined by the thiobarbituric acid method (Heath & Packer, 1968). Fresh samples were cut into small pieces and ground in liquid nitrogen with a mortar and pestle. The enzyme was extracted by phosphate-buffered saline (PBS, pH 7.0) solution containing ethylenediaminetetraacetic acid (1 mmol·L^−1^) and 1% polyvinylpyrrolidone. The homogenate was centrifuged at 15,000 g for 20 min at 4 °C, and the supernatant was collected to measure enzyme activity. Superoxide dismutase (SOD) activity was assayed based on the capacity to inhibit the photochemical reduction of nitro blue tetrazolium, and the absorbance was determined at 560 nm (Giannopolitis & Ries, 1977). Peroxidase (POD) activity was assayed by the guaiacol method, and the absorbance was determined at 470 nm (Chance & Maehly, 1955). Catalase (CAT) activity was detected based on the disappearance of hydrogen peroxide, and the absorbance was determined at 240 nm (modified from Verma & Dubey (2003)). Ascorbate peroxidase (APX) activity was assayed by a decrease in the absorbance of ascorbate, and the absorbance was determined at 290 nm (modified from Vanacker, Carver & Foyer (1998)).

### Observation under light microscopy and scanning electron microscopy

To visualize the anatomical structures of the leaves, stems and lateral roots, samples were hydrated in an ethanol series for 30 min at each step, cleared with dimethylbenzene for 15 min and then embedded in paraffin. Thereafter, thin sections of 3–4 μm thickness were fixed on microscope slides and then stained with Safranin O (Servicebio, Wuhan, China) and Fast Green (Servicebio, Wuhan, China) as described by Yoshida & Shirasu (2009). Sections were observed with a bright-field microscope (Leica DM6B; Leica Microsystems, Germany).

For scanning electron microscopy (SEM), samples prefixed in 2.5% glutaraldehyde were post fixed in 1% osmium tetroxide in 0.1 mol·L^−1^ PBS (pH 7.4) for 2 h at 20 °C. The segments were washed three times with 0.1 mol·L^−1^ PBS for 15 min each time and then dehydrated in a series of ethanol treatments (30%, 50%, 70%, 80%, 90%, 95%, 100% and 100%; each step for 15 min). The sample was treated with isopentyl acetate for 15 min, dried with a critical-point dryer (Quorum K850; Quorum Technologies Ltd., Lewes, UK), coated with gold using an ion sputter (IXRF MSP-2S) as described by Cui et al. (2016), and then observed and photographed with a scanning electron microscope (Hitachi SU8100, Hitachi High-Technologies, Japan). All treatments from three independent replicates (every independent replicate generated six sections) were observed. In addition, the stele diameter was measured manually from two cross-sections for each root segment using ImageJ. The SEM images were used to quantify the stomatal density on leaf abaxial surfaces. The number of stomata were counted from two images for each leave, and each image was taken from different leaf zones (Kiani-Pouya et al., 2020). The surface area of each leaf zone was measured using ImageJ. The stomatal density (number of stomata per unit of area) was then calculated.

### Statistical analysis

UNIANOVA (general linear model, univariate) was used to analyze the data of growth traits, physiological performance and structure-related characteristics, with the two-level factor “host” (host presence, host absence) and two-level factor “growth phase” (PE phase, AE phase) as factors. Univariate analysis of variance (ANOVA) was used on the data of studied traits to determine the significance of differences between tested treatments, and the means of the treatments were compared by Duncan’s multiple range tests at a 0.05 probability level. All the statistical analyses were performed using IBM Statistical Product and Service Solutions software (version 22; IBM Inc., Endicott, NY, USA).

## Results

### Positive effects of the establishment of the parasite-host association on growth indicators in *M. savatieri* seedlings

**Figure 1:**
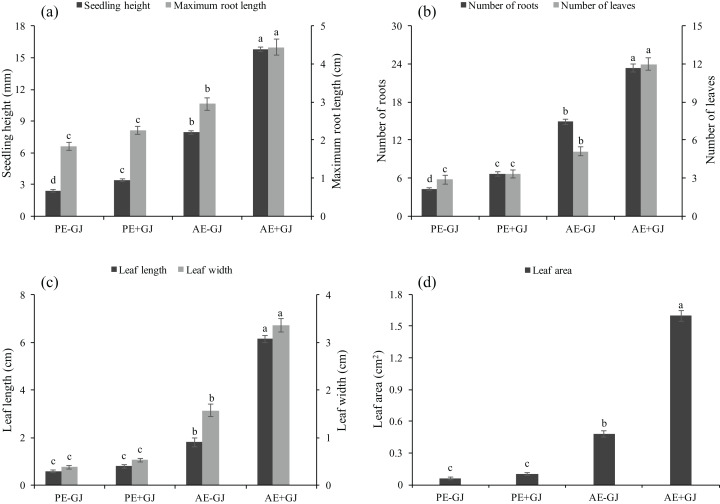
Seedling height, maximum root length (A), number of roots, leaves (B), leaf length, leaf width (C) and leaf area (D) of *M. savatieri* grown with or without a host after 8 and 16 weeks of sowing. Data are presented as the mean ± standard error of nine replicates. Different letters for the same indicator indicate statistically significant differences (*P* < 0.05). Treatments: PE−GJ, *M. savatieri* growing without a host 8 weeks after sowing; PE+GJ, *M. savatieri* growing with one *G. jasminoides* plant 8 weeks after sowing; AE−GJ, *M. savatieri* growing without a host 16 weeks after sowing; AE+GJ, *M. savatieri* growing with one *G. jasminoides* plant 16 weeks after sowing.

Significant differences were observed between the two key stages of *M. savatieri* development, that is, before and after the establishment of the *M. savatieri*–*G. jasminoides* association, in our experiment (Table S1). As shown in Fig. S1, attachment to a host significantly promoted the growth and development of *M. savatieri*, whereas seedlings grown without a host displayed relatively consistent growth and development in the two stages. Prior to the establishment of the parasite-host association, the RL, L, LL, LW and LA of *M. savatieri* were not significantly different between seedlings grown with or without a host (Fig. 1). The SH and R were significantly increased by the presence of a host. In the “AE” phase, *M. savatieri* parasitizing *G. jasminoides* (Figs. S1B and S1C) showed significantly greater SH, RL, R, L, LL, LW and LA than *M. savatieri* without a parasite-host association (Fig. 1).

### Establishment of the parasite-host association increased haustorium formation and biomass in *M. savatieri* seedlings

**Figure 2:**
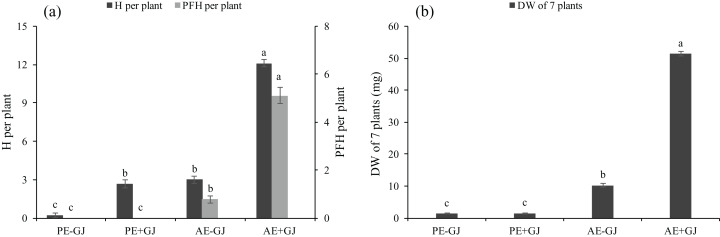
Number of haustoria (H) and presumably functional haustoria (PFH) per *M. savatieri* (A) and dry weight (DW) of seven *M. savatieri* plants (B) grown with or without a host after 8 and 16 weeks of sowing. Data are presented as the mean ± standard error (nine replicates for H and PFH per plant; six replicates for DW of seven plants). Different letters for the same indicator indicate statistically significant differences (*P* < 0.05). Treatments: PE−GJ, *M. savatieri* growing without a host 8 weeks after sowing; PE+GJ, *M. savatieri* growing with one *G. jasminoides* plant 8 weeks after sowing; AE−GJ, *M. savatieri* growing without a host 16 weeks after sowing; AE+GJ, *M. savatieri* growing with one *G. jasminoides* plant 16 weeks after sowing.

*M. savatieri* formed haustoria when grown without a host (Fig. S1D; Fig. 2A). The presence of *G. jasminoides* showed no significant effects on the total DW of *M. savatieri* in the “PE” phase (Fig. 2B). The H in *M. savatieri* was significantly higher when grown with a host than when grown without a host. In the “AE” phase, *M. savatieri* seedlings that established parasite-host associations produced significantly more H and PFH than those grown without a host (Fig. 2A). When grown with *G. jasminoides*, the DW of the seedlings after the establishment of parasite-host associations increased by 33.6 times compared with that of the corresponding seedlings before the establishment of parasite-host associations. In the absence of a host, the DW of the seedlings in the “AE” phase increased by 7.7 times compared with that of the corresponding seedlings in the “PE” phase. Moreover, the DW of *M. savatieri* that established parasite-host associations was 402.25% higher than that of seedlings grown without hosts in the “AE” phase (Fig. 2B).

### Accumulation of chlorophyll content in seedlings with established parasite-host association

**Figure 3:**
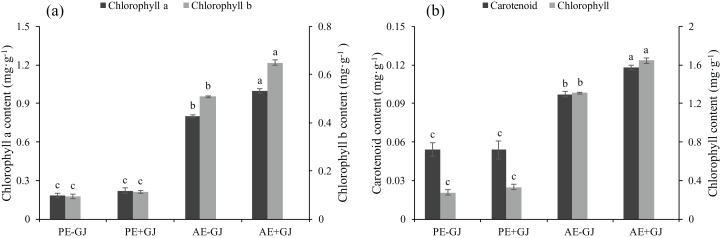
Chlorophyll a content, chlorophyll b content (a), carotenoid content, and chlorophyll content (b) of *M. savatieri* grown with or without a host after 8 and 16 weeks of sowing. Data are presented as the mean ± standard error of three replicates. Different letters for the same indicator indicate statistically significant differences (*P* < 0.05). Treatments: PE−GJ, *M. savatieri* growing without a host 8 weeks after sowing; PE+GJ, *M. savatieri* growing with one *G. jasminoides* plant 8 weeks after sowing; AE−GJ, *M. savatieri* growing without a host 16 weeks after sowing; AE+GJ, *M. savatieri* growing with one *G. jasminoides* plant 16 weeks after sowing.

Prior to the establishment of the parasite-host association, the presence of *G. jasminoides* had no significant effects on the chl a, chl b, car or chlorophyll contents of *M. savatieri* (Fig. 3). However, as the growth period continued, the chl a, chl b, car and chlorophyll contents of *M. savatieri* seedlings grown both with and without *G. jasminoides* increased. In the “AE” phase, the establishment of the parasite-host association significantly increased the photosynthetic pigment contents of *M. savatieri*, and the maximum values of chl a, chl b, car and chlorophyll contents were all observed in the seedlings connected with hosts.

### Establishment of the parasite-host association increased the contents of TSS, SP and proline and decreased CMP

**Figure 4:**
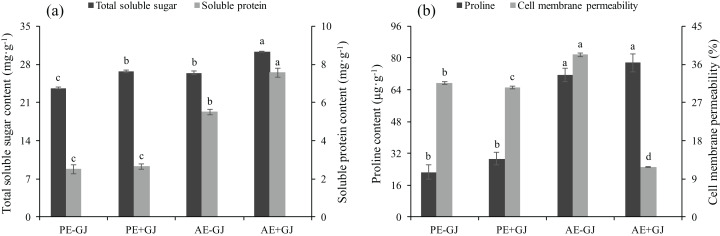
Total soluble sugar content, soluble protein content (A), proline content and cell membrane permeability (B) of *M. savatieri* grown with or without a host after 8 and 16 weeks of sowing. Data are presented as the mean ± standard error of three replicates. Different letters for the same indicator indicate statistically significant differences (*P* < 0.05). Treatments: PE−GJ, *M. savatieri* growing without a host 8 weeks after sowing; PE+GJ, *M. savatieri* growing with one *G. jasminoides* plant 8 weeks after sowing; AE−GJ, *M. savatieri* growing without a host 16 weeks after sowing; AE+GJ, *M. savatieri* growing with one *G. jasminoides* plant 16 weeks after sowing.

Both the host presence and developmental phase had significant effects on the contents of TSS and SP of *M. savatieri* (Table S2). The contents of TSS and SP were highest in the *M. savatieri* seedlings connected with *G. jasminoides* (Fig. 4A). In the “PE” phase, the SP content in seedlings was not significantly different in seedlings grown with or without a host, while seedlings parasitizing a host accumulated significantly more SP in the “AE” phase. When grown with *G. jasminoides*, the TSS content in seedlings before and after the establishment of the parasite-host associations was significantly greater than that in seedlings grown without a host, increasing by 13.04% and 14.76%, respectively.

The proline content in seedlings in the “AE” phase was significantly higher than that in the “PE” phase, independent of the presence of a host (Fig. 4B). In contrast, the cell membrane permeability (CMP) of *M. savatieri* showed significant differences between the tested treatments, and the minimum value of CMP was observed in the seedlings that established parasite-host associations. When grown without a host, the value of CMP in the seedlings significantly increased with increasing growth time.

### Establishment of the parasite-host association increased the activities of antioxidant enzymes and attenuated MDA accumulation in *M. savatieri* seedlings

**Figure 5:**
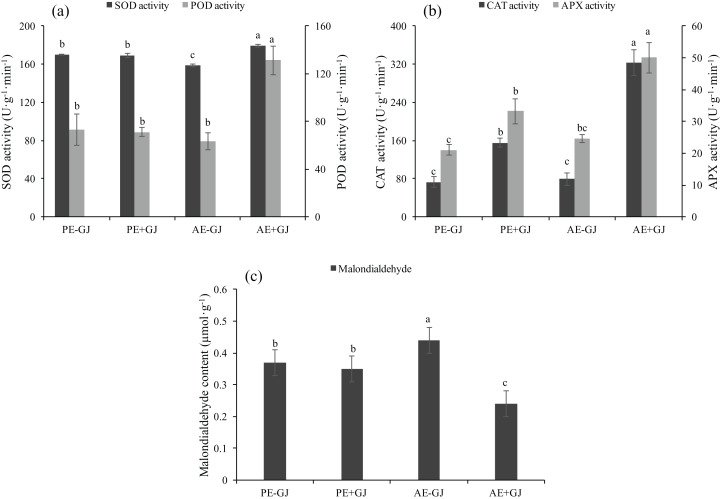
SOD activity, POD activity (A), CAT activity, APX activity (B) and malondialdehyde content (C) of *M. savatieri* grown with or without a host after 8 and 16 weeks of sowing. Data are presented as the mean ± standard error of three replicates. Different letters for the same indicator indicate statistically significant differences (*P* < 0.05). Treatments: PE−GJ, *M. savatieri* growing without a host 8 weeks after sowing; PE+GJ, *M. savatieri* growing with one *G. jasminoides* plant 8 weeks after sowing; AE−GJ, *M. savatieri* growing without a host 16 weeks after sowing; AE+GJ, *M. savatieri* growing with one *G. jasminoides* plant 16 weeks after sowing.

When grown with *G. jasminoides*, the MDA content of *M. savatieri* measured in the “AE” phase was significantly lower than that of seedlings grown without a host, whereas the presence of a host had no significant effects on the MDA content of seedlings measured in the “PE” phase (Fig. 5C). Prior to the establishment of the parasite-host associations, the seedlings grown with a host exhibited significantly higher activities of CAT and APX than those grown without a host (Fig. 5B). In the “AE” phase, the establishment of the parasite-host associations significantly increased the activities of SOD, POD, CAT and APX in *M. savatieri* seedlings compared with those in seedlings grown without a host (Figs. 5A and 5B).

### Development characteristics of leaves in *M. savatieri* seedlings

Under the four sampling conditions, the upper and lower epidermis of *M. savatieri* leaves contained only one layer of cells, and the epidermal cells of seedlings grown with *G. jasminoides* were smaller than those of seedlings grown without a host (Figs. S2A–S2D). The isobilateral leaves of *M. savatieri* were observed, and the mesophyll cells were irregular in shape and abundant in chloroplasts. Moreover, more chloroplasts were found in the leaves of seedlings sampled in the “AE” phase than in those sampled in the “PE” phase. A type of collateral vascular bundle was found in the veins of the seedlings. After invading the host, the vascular bundles of *M. savatieri* leaves developed well, especially the xylem in the midrib (Fig. S2D). For a detailed characterization, we observed the leaves of *M. savatieri* with SEM (Figs. S2E–S2J). The epidermis of the *M. savatieri* leaves was uneven and closely fitted, showing a ridged structure (Fig. S2E) with many stomata scattered on the epidermis (Fig. S2F). Growth phase had a significant effect on stomatal density of *M. savatieri* (Table S3), with “PE” phase showed significant higher stomatal density than “AE” phase, regardless of the presence of a host (Table S4). Non-glandular epidermal hairs and glandular trichomes were both observed on the leaves of the seedlings. The non-glandular epidermal hairs were the tip-curved papillary type and were observed on the leaf surface (Fig. S2G), the leaf edge (Fig. S2H) and the petioles (Fig. S2I). However, glandular trichomes with multicellular uniseriate stalks and unicellular heads were observed on the leaf vein (Fig. S2J), the leaf edge (Fig. S2H) and the petioles (Fig. S2I). In addition, the leaves of the seedlings invading the host had many more non-glandular epidermal hairs and glandular trichomes than the leaves of the other seedlings (Fig. S3).

### Development characteristics of stems in *M. savatieri* seedlings

When *M. savatieri* seedlings established parasite-host associations, the SH of *M. savatieri* increased sharply (Fig. 1; Fig. S1). To understand the early events in *M. savatieri* stem development, we carefully examined the anatomical structures of *M. savatieri* stems before and after establishment of the parasite-host association. In the “PE” phase, the stems of seedlings grown with or without *G. jasminoides* were characterized by few stomata, multicellular epidermal hairs and small vascular bundles (Figs. S4A and S4B). In the “AE” phase, more than half of the vascular bundle of seedlings grown without the host was composed of xylem tissue, and the remainder was composed of phloem tissue (Fig. S4C). After establishing parasite-host associations, the stems of *M. savatieri* seedlings exhibited a typical primary structure (Fig. S4D) of the medulla and medullary ray in the vascular cylinder; the collateral vascular bundle was arranged in a circle, and the elliptical parenchyma cells constituting the medulla were large and loosely arranged. Moreover, a large number of epidermal hairs were also observed in the stems of *M. savatieri* seedlings that had established parasite-host associations.

### Development characteristics of roots in *M. savatieri* seedlings

**Figure 6:**
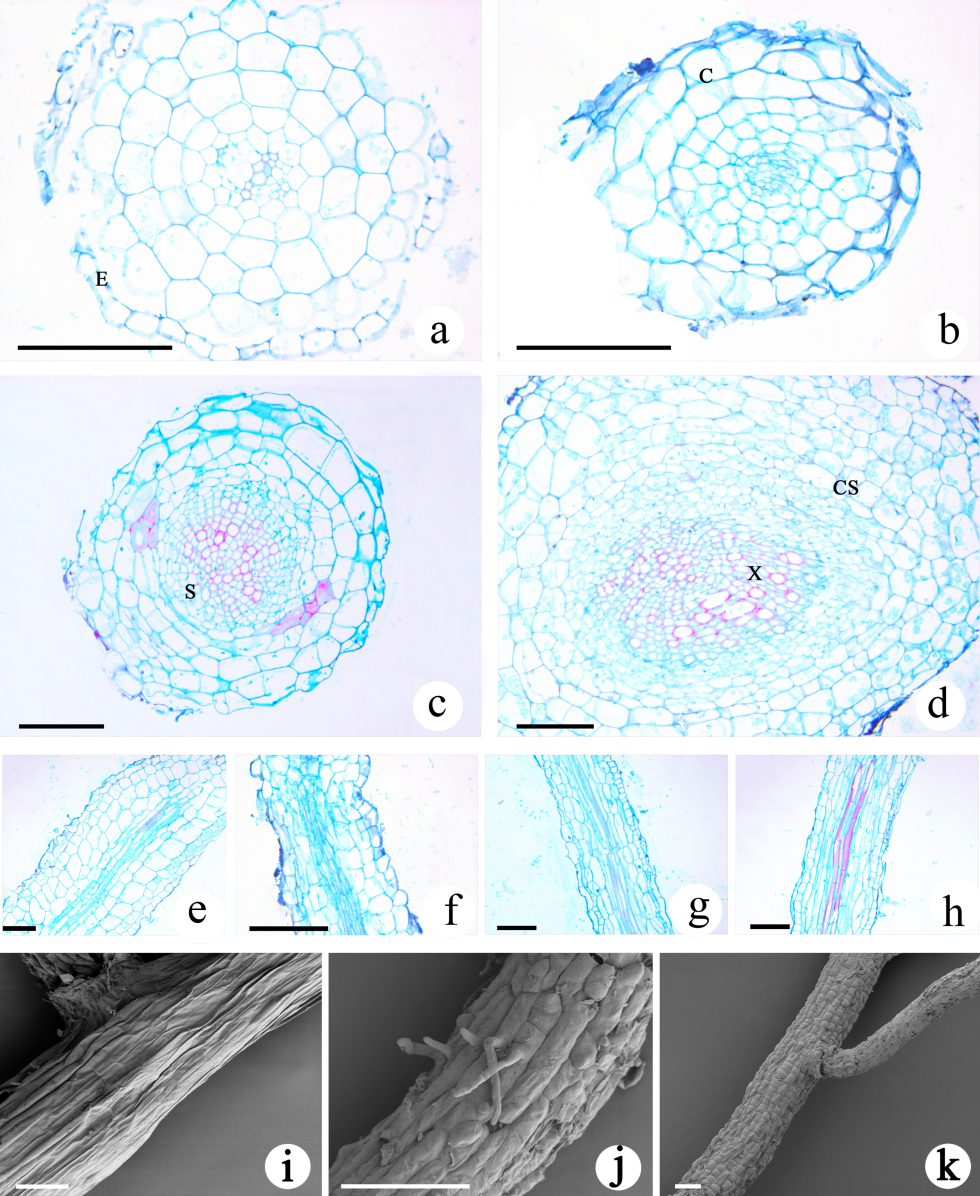
Microscopic examination of *M. savatieri* roots. (A–D) Cross-sections of *M. savatieri* roots. (E–H) Longitudinal sections of *M. savatieri* roots. (A) and (E) show *M. savatieri* growing without a host 8 weeks after sowing (PE−GJ). (B) and (F) show *M. savatieri* growing with one *G. jasminoides* plant 8 weeks after sowing (PE+GJ). (C) and (G) show *M. savatieri* growing without a host 16 weeks after sowing (AE−GJ). (D) and (H) show *M. savatieri* growing with one *G. jasminoides* plant 16 weeks after sowing (AE+GJ). (I–K) SEM images of *M. savatieri* roots. Note that the formation of the lateral root ruptured the epidermis of the parent root. C, cortex; CS, Casparian strip; E, epidermis; S, stele; X, xylem. Bars 100 μm.

The transverse section of the roots of *M. savatieri* seedlings was nearly circular, and the young roots examined during this study consisted of the epidermis, cortex and stele (Figs. 6A–6D). In comparison with those in seedlings grown without *G. jasminoides*, the cells making up the cortex parenchyma formed more layers and were arranged more compactly in the roots of *M. savatieri* seedlings that had established parasite-host associations. The size of the stele differed between the four sampling conditions and was better developed in the roots of seedlings that had parasitized a host. Variations in host presence and growth phase showed significant effects on the stele diameter (Table S3). In the “AE” phase, stele diameter of *M. savatieri* roots was substantially increased when attached to a host (Table S4). The primary xylem was barely visible in the roots of seedlings sampled in the “PE” phase, while the primary xylem accounted for half of the stele of the roots in seedlings sampled in the “AE” phase (Figs. 6A–6D). The longitudinal structures of *M. savatieri* roots are shown in Figs. 6E–6H. Among all examined roots, the xylem, with Safranin O-stained (reddish) lignified cell walls, was observed distinctly in the roots of seedlings after the establishment of the parasite-host associations. SEM was utilized to further view the surface of *M. savatieri* roots, which bore root hairs that were lateral extensions of a single cell, without branching (Fig. 6J). The cells that formed a long strip and a scale shield were arranged compactly to make up the surface of *M. savatieri* roots. (Figs. 6I–6K).

### Analysis of haustoria development in *M. savatieri* seedlings

**Figure 7:**
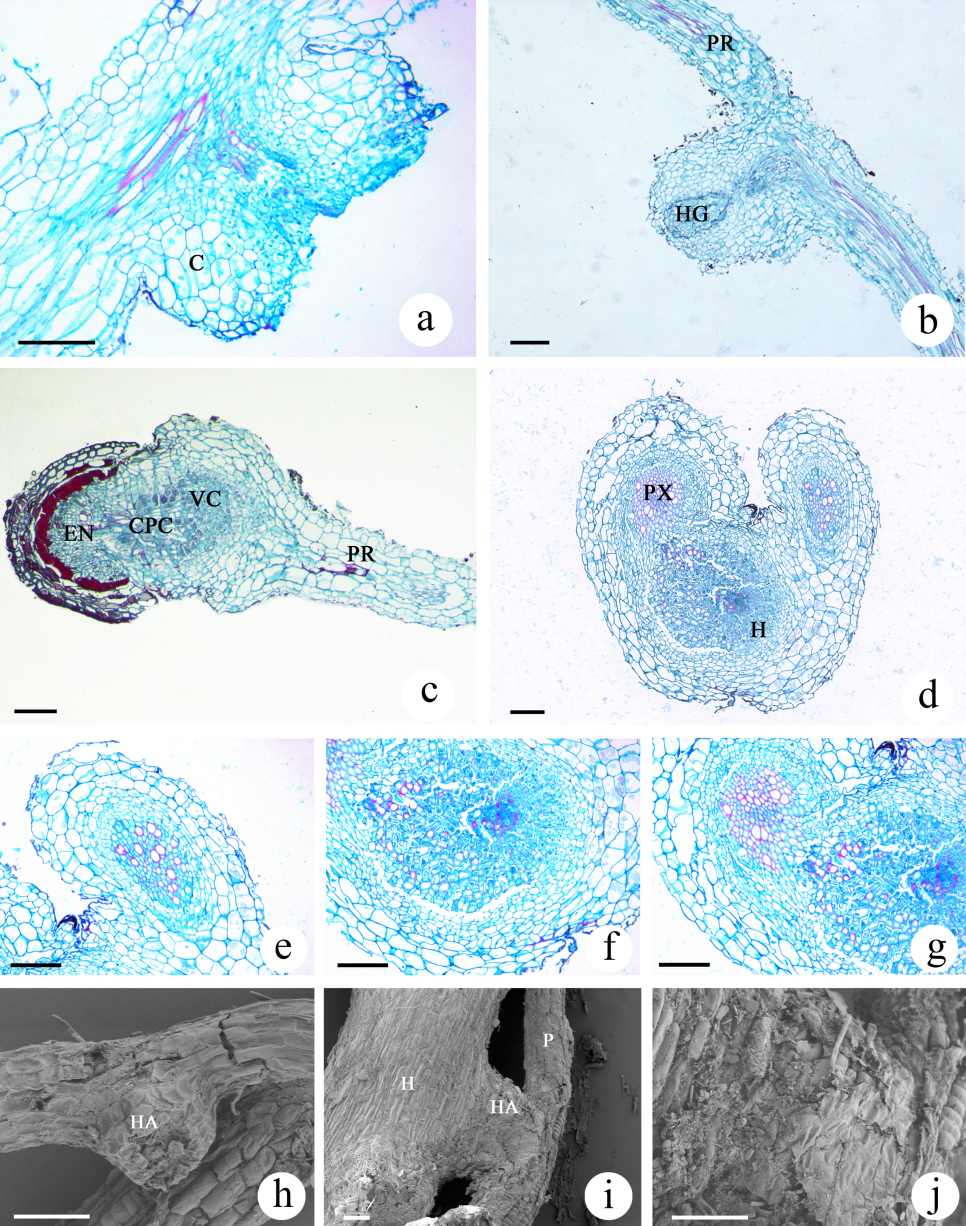
Microscopic examination of *M. savatieri* haustoria. (A–C) Longitudinal sections of *M. savatieri* haustoria. (A) Two young haustoria formed next to each other on a single *M. savatieri* root. (B) A haustorium that was close to forming a xylem bridge. Note that the host root was broken during sampling. (C) *M. savatieri* connected with *G. jasminoides* roots. (D–G) Cross-sections of *M. savatieri* infecting *G. jasminoides*. (D) The two stages during the parasite infection of the host. (E) The early stage of the parasite in contact with the host. (F) A detailed view of the host root consisting of parasitic xylem. (G) The parasite xylem connected with the host xylem. (H–J) SEM images of *M. savatieri* haustoria. (H) A haustorium that was detached from the host roots. (I) A haustorium of *M. savatieri* attached to the host root. (J) A magnified photo of the junction between the haustorium and the host root. (A–J) Show *M. savatieri* growing with one *G. jasminoides* plant 16 weeks after sowing. C, cortex; CPC, central parenchymatous core; E, epidermis; EN, endophyte; H, host; HA, haustorium; HG, haustorial gland; HX, host xylem; P, parasite; PR, parent root; PX, parasite xylem; VC, vascular core. Bars 100 μm.

The haustoria developed by *M. savatieri* were globular or hemispherical and were the lateral type of haustoria, which form from the lateral roots of parasites (Fig. S1D; Figs. 7A and 7B). The lateral haustoria formed by *M. savatieri* are referred to as haustoria in this article. The haustoria of *M. savatieri* were small, with a diameter of less than 0.5 mm. During the haustorium development of *M. savatieri*, the earliest events in the growth of the anatomical structures were the expansion of cortical cells and cell division in the epidermis. Interestingly, *M. savatieri* formed two haustoria on a single root because the lateral haustoria did not hinder the meristematic activity of the roots (Fig. 7A). After the young haustorium came into contact with *G. jasminoides*, the cells from the epidermis at the tip of the haustorium began to divide and elongate (Fig. 7E). Concomitant with the penetration of the haustorium, the cell walls and cell membranes of the host root at the parasite-host interface disintegrated and degraded (Figs. 7D and 7G). Subsequently, a unique region called the internal cavity or the haustorial gland developed in the center of the haustorium and extended toward the haustorium-host junction. At the same stage of development, some vessels or tracheary elements that were differentiated from the cells at the center of the haustorium were detected in the internal cavity or haustorial gland and could be developed into a xylem bridge. In addition, haustorial cells close to the parasite stele differentiated into xylem cells and proceeded toward the center of the haustorium (Fig. 7B). When parasite-host vascular continuity was established, the completely mature haustoria of *M. savatieri* consisted of three distinct portions: the central parenchymatous core, the vessels and the endophyte. The central parenchymatous core, containing a large amount of deposited substances, was connected to the vascular core of the parasite and was located in the center of the haustorium. The vessels were oriented toward the host xylem (Fig. 7C). In the endophyte, the xylem cells were accompanied by parenchyma cells. The parenchyma cells in the endophyte were characterized by dense protoplasmic contents and an elongated shape, and they extended between the host cells and proceeded toward the host vascular tissue (Fig. 7C and 7F). The SEM studies further broadened our understanding of *M. savatieri* haustoria. The haustorium was approximately the same width as the parent root or slightly wider than the parent root. When *M. savatieri* penetrated into the host root, the point of entry of the endophyte was not visible on the parasite-host interface because the parasite formed a broad collar (Fig. 7I). However, a detached haustorium examined by SEM clearly showed an opening on the surface of the haustorium (Fig. 7H). Fragmentation was observed on the surface of the haustorium attached to the host root (Fig. 7J).

## Discussion

The establishment of the parasite-host association is the essence of the parasitic plant life habit and is considered an important developmental step in the life cycle of hemiparasites in Orobanchaceae (Joel, 2013). The consequences of the establishment of parasite-host associations for many hemiparasites, such as *Thesium chinense and Pedicularis rex*, have been studied extensively (Guo & Luo, 2010; Li et al., 2012). However, similar reports are lacking for *M. savatieri*. Given that *M. savatieri* has great potential for the medical industry and that its wild resources have declined sharply in recent years (Shi et al., 2013; Zhang et al., 2015), the growth of *M. savatieri* before and after the establishment of the parasite-host association was evaluated in this study. Furthermore, data on the physiology and anatomical structure of *M. savatieri* in these two developmental periods were provided and discussed.

### Establishment of the parasite-host association stimulated seedling growth in *M. savatieri*

In the “AE” phase, establishing parasite-host associations markedly promoted the growth of *M. savatieri* not only in terms of the SH, RL, R, L, LL, LW and LA (Fig. 1) but also in terms of the total DW, H and PFH (Fig. 2) compared with those of seedlings grown without *G. jasminoides*. The results clearly demonstrated that *M. savatieri* seedlings attached to a host showed better growth and development. Similar growth promotion has been reported in *T. chinense* that have established associations with their preferred hosts (Guo & Luo, 2010). These results may be partially explained by the weak capacity for autotrophic growth as a result of the vestigial root system of the parasites (Li et al., 2012); water and nutrients are transferred from hosts to parasites through the attached haustoria (Westwood et al., 2010). On the other hand, symptoms of slow growth occurred in *M. savatieri* grown without a host, but the parasite survived for at least 16 weeks, which indicated that establishment of the parasite-host association is not necessary to *M. savatieri* for survival but is necessary for its better development. Under potted conditions, the seedlings experienced limited space and P supply in the soil; therefore, competition with the host played an important role in the parasite-host association (Keith, Cameron & Seel, 2004; Matthies, 1995). In this study, *G. jasminoides* plants were planted in the pots one month before sowing. The amount of substrate nutrients at sowing time differed between the pots with and without hosts, as did the light availability. These factors may result negative early effects of the host presence. However, prior to the establishment of the parasite-host association, the host presence had no negative effects on the RL, leaf indexes and DW of *M. savatieri*, and had even positive effects on the SH, R and H of *M. savatieri*. The main reason might be that although had not successfully parasitized on the host in the “PE” phase, *M. savatieri* had an early contact to the host. The phase “prior to establishment of the parasite-host association” might not only include “prior to establishment”, but also “during early establishment”.

Root hemiparasites commonly have large host ranges (Irving & Cameron, 2009), and the similar phenomenon has been observed in *M. savatieri* (Zhang et al., 2015). A range of hosts may supply the parasites with varied amounts and types of nutrients, thereby providing an ecological advantage (Govier, Nelson & Pate, 1967). The host range is mainly affected by the relationship between ecology (environmental factors and dispersal biology) and geography (host distribution) (Heide-Jørgensen, 2013). The hosts of *M. savatieri* are characterized by the shallow and multi-branched root systems, and small leaves and branches, which suggested that *M. savatieri* exhibits a host preference (Zhang et al., 2015). In this experiment, we reported the growth promotion when *M. savatieri* attached to *G. jasminoides* (Figs. 1 and 2). How would *gardenia* compare to other genera as a host? Since host preference has been studied for some hemiparasitic species, regardless of their degree of parasitism (Guo & Luo, 2010; Ren et al., 2010; Li et al., 2012). It was concluded that the probability of being parasitized, developmental stages and competitive interaction may together determine host suitability. Nevertheless, further studies are needed to explore preference of *M. savatieri* in different growth stages according to the performance of the hemiparasite growing with different combinations of hosts.

### Establishment of the parasite-host association enhanced the autotrophic and adaptive capacity of *M. savatieri*

Our results demonstrated that establishing parasite-host associations resulted in a marked increase in the contents of photosynthetic pigments compared with those in the seedlings without a parasite-host association (Fig. 3). This suggests that the nutrients obtained from the host may have increased the contents of photosynthetic pigments in *M. savatieri*. Similarly, the chlorophyll contents of *Pedicularis kansuensis* attached to a host were threefold those of unattached *P. kansuensis* (Sui et al., 2015). These results may be related to the Rubisco content and cytochrome *f* content of the parasites and subsequently to their enhanced photosynthetic ability (Evans, 2013; Feng et al., 2012; Riahinia et al., 2013). Higher amounts of TSS, SP and proline were observed in *M. savatieri* seedlings that were connected with a host (Fig. 4), which may have supported these seedlings in maintaining an appropriate water potential and thus sustaining rapid growth (Figs. 1 and 2). Moreover, the greater accumulations of TSS and SP could meet the demands of vigorous metabolic activities. In contrast, *M. savatieri* without a parasite-host association uses its limited energy for slow growth and development only during a certain period, suggesting that the enhanced growth and development of *M. savatieri* was highly dependent on the host.

Compared with those of *M. savatieri* seedlings in the “PE” phase, the MDA and CMP values of seedlings in the “AE” phase were significantly different. The values increased in seedlings grown without a host but decreased in seedlings grown with a host (Figs. 4B and 5C). Oxidative damage induced by stress affects plants; therefore, they have evolved antioxidative defense mechanisms, such as the non-enzymatic and enzymatic processing systems, to overcome the oxidative stress caused by reactive oxygen species (ROS) (Das & Roychoudhury, 2014; Zagorchev, Teofanova & Odjakova, 2016). However, insufficient information was found in the literature concerning the reactions of the non-enzymatic and enzymatic antioxidants of parasitic plants to adverse environmental factors. In contrast, the protective roles of CAT, APX, POD and SOD against water and heat stress have been well investigated in many autotrophic species, such as alfalfa (*Medicago sativa* L.) (Salemi, Esfahania & Tran, 2019) and *Melissa officinalis* (Pistelli et al., 2019). The results of the current study revealed significantly increased activities of the antioxidant enzymes SOD, CAT, POD, and APX in *M. savatieri* seedlings that were connected with the host compared with those in seedlings with no parasite-host associations (Figs. 5A and 5B). The increased activities of these antioxidant enzymes seemed to have reached levels that could balance the excessive ROS, which was shown by the lower MDA content detected in the *M. savatieri* seedlings connected with a host (Fig. 5C). Among the non-enzymatic antioxidants, car play a significant role in plant metabolic activities, such as photoprotection, ROS scavenging, and protection of membrane lipids from peroxidative damage (Havaux, 1998). In this study, the highest car content was observed in seedlings after the establishment of parasite-host associations, suggesting that connecting with the host may have allowed *M. savatieri* to produce enough metabolites to reduce ROS generation and protect the thylakoid membranes. Taken together, these results suggest that the establishment of parasite-host associations may enable *M. savatieri* to enhance its autotrophic capacity and stress tolerance, thus resulting in better growth and development. Further studies are needed to investigate the mechanisms by which specific environmental factors affect *M. savatieri*, which would assist in establishing a comprehensive cultivation system for the better preservation and exploitation of *M. savatieri*.

### Establishment of the parasite-host association promoted the structural development of *M. savatieri*

*M. savatieri* leaves consisted of isobilateral mesophyll tissue, collateral vascular bundles, and stomata located on both the adaxial and abaxial surfaces (Figs. S2A–S2D). Moreover, the leaves had chloroplasts and were capable of photosynthesis; these characteristics are similar to those of three species in the parasitic Rhinantheae tribe (Kaplan & Inceoglu, 2003). In the “AE” phase, the leaves of *M. savatieri* seedlings that established parasite-host associations showed better-developed vascular bundles and a larger number of chloroplasts than those without a parasite-host association. This result might provide a partial explanation for the larger leaf indexes and photosynthetic pigment contents in seedlings that established parasite-host associations. Both glandular and non-glandular trichomes were observed on the leaves of *M. savatieri* seedlings (Figs. S2G–S2J). Glandular trichomes are involved in accumulating salt and protecting plants from pathogens and herbivores (Werker, 2000). Non-glandular trichomes contribute to increased tolerance of drought, high temperatures and freezing, and offer protection against UV light and insect herbivores (Johnson, 1975; Mauricio & Rausher, 1997). As wild *M. savatieri* seedlings grow on the sunny slopes of small mountains and hills, it is possible that *M. savatieri* seedlings developed a larger number of non-glandular trichomes after establishing parasite-host associations to adapt to the sunlight and reduce their water loss.

Interestingly, the examined *M. savatieri* roots were all in the primary growth stage (Figs. 6A–6D). This suggests that primary growth may represent an opportunity for the roots of hemiparasites to increase in length to search for available water, nutrients and host roots, even if they have attached the host. The root cortex plays a role in starch storage (Crang, Lyons-Sobaski & Wise, 2018), and the much larger root cortex of the attached *M. savatieri* seedlings at the same phase than in the unattached seedlings suggests that the nutrients obtained from the host likely allowed the hemiparasite to accumulate more starch, thus providing energy for further root development and seedling growth (Figs. 6A–6H). During the establishment of parasite-host associations, the development of mature haustoria is fundamental and involves dynamic changes in structure (Yoshida et al., 2016). Distinct structural shifts in the haustoria of *M. savatieri*, such as the swelling of the haustorium-forming site of the root (Fig. 7A), the formation of the internal cavity or haustorial gland in the inner haustorium (Fig. 7B), and the vascular connection between the parasite and the host (Figs. 7C and 7D), were observed. The haustorial gland is considered a unique feature of the haustorium in Santalaceae (Kuijt, 1969) and regularly develops in the interior of the haustorium in *Santalum album* after the haustorium comes into contact with the host root (Zhang et al., 2012). In the current study, the haustorial gland was observed in the haustorium of *M. savatieri*, suggesting that the haustoria of Orobanchaceae may also develop haustorial glands, although no other similar studies have documented this characteristic.

## Conclusions

Changes in the growth traits, physiological performance and anatomical structures of parasitic plants before and after the establishment of parasite-host associations were systematically studied for the first time. Our results demonstrated that the establishment of parasite-host associations substantially promoted the growth and development of *M. savatieri* and stimulated antioxidative defense mechanisms in the seedlings. Prior to the establishment of the parasite-host associations, the presence of the host had no significant effect on the maximum RL, leaf indexes or total DW, but had significant positive effect on SH, Rs or H. Future work needs to take different hosts into account for the development of artificial cultivation systems for *M. savatieri*. Studies with combined advanced technologies such as transcriptomics and metabolomics will increase our understanding of the regulatory mechanisms driving the establishment of parasite-host associations.

## Supplemental Information

10.7717/peerj.9780/supp-1 Supplemental Information 1 Summary of UNIANOVA (general linear model, univariate) results (*F*-values and significance levels) for the effects of host and growth phase on growth traits of *M. savatieri*. **The level of significance is *P* < 0.01. df, degrees of freedom. SH, seedling height; RL, maximum root length; R, number of roots; L, number of leaves; LL, leaf length; LW, leaf width; LA, leaf area; H, number of haustoria; PFH, number of presumably functional haustoria; DW, dry weight.

10.7717/peerj.9780/supp-2 Supplemental Information 2 Summary of UNIANOVA (general linear model, univariate) results (*F*-values and significance levels) for the effects of host and growth phase on physiological performance of *M. savatieri*. *The level of significance is *P* < 0.05. **The level of significance is *P* < 0.01. df, degrees of freedom. chl a, chlorophyll a; chl b, chlorophyll b; car, carotenoid; TSS, total soluble sugar; SP, soluble protein; P, proline; CMP, cell membrane permeability; MDA, malondialdehyde; SOD, superoxidedismutase; POD, peroxidase; CAT, catalase; APX, ascorbate peroxidase.

10.7717/peerj.9780/supp-3 Supplemental Information 3 Summary of UNIANOVA (general linear model, univariate) results (*F*-values and significance levels) for the effects of host and growth phase on root stele diameter and stomatal density of *M. savatieri*. ** The level of significance is *P* < 0.01. df, degrees of freedom.

10.7717/peerj.9780/supp-4 Supplemental Information 4 Root stele diameter and stomatal density of *M. savatieri* plants grown with or without a host after 8 and 16 weeks of sowing. Data are presented as the mean ± standard error (*n* = 6). Different letters for the same indicator indicate statistically significant differences (*P* < 0.05). Treatments: PE−GJ, *M. savatieri* growing without a host 8 weeks after sowing; PE+GJ, *M. savatieri* growing with one *G. jasminoides* plant 8 weeks after sowing; AE−GJ, *M. savatieri* growing without a host 16 weeks after sowing; AE+GJ, *M. savatieri* growing with one *G. jasminoides* plant 16 weeks after sowing.

10.7717/peerj.9780/supp-5 Supplemental Information 5 Seedling growth and haustoria development of *M. savatieri*. (A) PE−GJ, *M. savatieri* growing without a host 8 weeks after sowing; PE+GJ, *M. savatieri* growing with one *G. jasminoides* plant 8 weeks after sowing; AE−GJ, *M. savatieri* growing without a host 16 weeks after sowing; AE+GJ, *M. savatieri* growing with one *G. jasminoides* plant 16 weeks after sowing. (B) *M. savatieri* seedling (white arrow) parasitizing the roots of *G. jasminoides* (red arrow). (C) Micrograph of the haustorial connection between *M. savatieri* (white arrow) and *G. jasminoides* (red arrow). (D) Micrograph of the haustoria of *M. savatieri* (white arrow) in the absence of a host. (A) and (B) Bar 1 cm. (C) and (D) Bar 1 mm.

10.7717/peerj.9780/supp-6 Supplemental Information 6 Microscopic examination of *M. savatieri* leaves. (A–D) Cross-sections of *M. savatieri* leaves. Panel a shows *M. savatieri* growing without a host 8 weeks after sowing. (B) Shows *M. savatieri* growing with one *G. jasminoides* plant 8 weeks after sowing. (C) Shows *M. savatieri* growing without a host 16 weeks after sowing. (D) Shows *M. savatieri* growing with one *G. jasminoides* plant 16 weeks after sowing. (E–J) SEM images of *M. savatieri* leaves. (E) Shows that the epidermis of the leaves was uneven and closely fitted, showing a ridged-like structure. (F) Shows a stomata and two guard cells on the epidermis. (G) Shows that tip-curved papillary epidermal hairs occurred sparsely on the leaves. (H) Shows epidermal hairs and a glandular trichome distributed at the edge of the leaves. (I) Shows epidermal hairs and glandular trichomes distributed on the petioles. (J) Shows glandular trichomes distributed on the leaf vein. C, chloroplast; E, epidermis; M, mesophyll; S, stomata; X, xylem. Bars 100 μm.

10.7717/peerj.9780/supp-7 Supplemental Information 7 SEM images of *M. savatieri* leaves. (A) *M. savatieri* growing without a host 8 weeks after sowing (PE−GJ). (B) *M. savatieri* growing with one *G. jasminoides* plant 8 weeks after sowing (PE+GJ). (C) *M. savatieri* growing without a host 16 weeks after sowing (AE−GJ). (D) *M. savatieri* growing with one *G. jasminoides* plant 16 weeks after sowing (AE+GJ). Bars 1 mm.

10.7717/peerj.9780/supp-8 Supplemental Information 8 Transverse sections of *M. savatieri* stems. (A) *M. savatieri* growing without a host 8 weeks after sowing (PE−GJ). (B) *M. savatieri* growing with one *G. jasminoides* plant 8 weeks after sowing (PE+GJ). (C) *M. savatieri* growing without a host 16 weeks after sowing (AE−GJ). (D) *M. savatieri* growing with one *G. jasminoides* plant 16 weeks after sowing (AE+GJ). C, cortex; E, epidermis; EH, epidermal hairs; M, medulla; MR, medullary ray; P, phloem; S, stomata; X, xylem. Bars 100 μm.

10.7717/peerj.9780/supp-9 Supplemental Information 9 Biological repetition values, means and standard deviations. This was used to compare the growth indicators, physiological performance and structure-related characteristics of seedlings before and after establishment of parasite-host association.

## Additional Information and Declarations

### Data Availability

The following information was supplied regarding data availability: The raw measurements are available in the Supplemental Files.
