# Supplementary material for: The changes of morphological and physiological characteristics in hemiparasitic Monochasma savatieri before and after attachment to the host plant
Source: PeerJ. 2020 Aug 19;8:e9780. doi: 10.7717/peerj.9780 (PMC7443084; doi:10.7717/peerj.9780)
Supplement: Supplemental Information 1 — **The level of significance is P < 0.01. df, degrees of freedom. SH, seedling height; RL, maximum root length; R, number of roots; L, number of leaves; LL, leaf length; LW, leaf width; LA, leaf area; H, number of haustoria; PFH, number of presumably functional haustoria; DW, dry weight. [file peerj-08-9780-s001.docx]

Table S1 Summary of UNIANOVA (general linear model, univariate) results (*F*-values and significance levels) for the effects of host and growth phase on growth traits of *M. savatieri*.

|  | df | SH | RL | R | L | LL | LW | LA | H | PFH | df | DW |
| --- | --- | --- | --- | --- | --- | --- | --- | --- | --- | --- | --- | --- |
| Host | 1, 32 | 760^**^ | 38^**^ | 157^**^ | 93^**^ | 323^**^ | 100^**^ | 404^**^ | 470^**^ | 130^**^ | 1, 20 | 2123^**^ |
| Growth phase | 1, 32 | 3106^**^ | 113^**^ | 988^**^ | 204^**^ | 675^**^ | 423^**^ | 1104^**^ | 526^**^ | 239^**^ | 1, 20 | 4282^**^ |
| Host × Growth phase | 1, 32 | 459^**^ | 12^**^ | 48^**^ | 72^**^ | 262^**^ | 71^**^ | 353^**^ | 157^**^ | 130^**^ | 1, 20 | 2079^**^ |

^**^ The level of significance is *P* < 0.01. df, degrees of freedom. SH, seedling height, RL, maximum root length, R, number of roots, L, number of leaves, LL, leaf length, LW, leaf width, LA, leaf area, H, number of haustoria, PFH, number of presumably functional haustoria, DW, dry weight.
