# Supplementary material for: The changes of morphological and physiological characteristics in hemiparasitic Monochasma savatieri before and after attachment to the host plant
Source: PeerJ. 2020 Aug 19;8:e9780. doi: 10.7717/peerj.9780 (PMC7443084; doi:10.7717/peerj.9780)
Supplement: Supplemental Information 2 — *The level of significance is P < 0.05. **The level of significance is P < 0.01. df, degrees of freedom. chl a, chlorophyll a; chl b, chlorophyll b; car, carotenoid; TSS, total soluble sugar; SP, soluble protein; P, proline; CMP, cell membrane permeability; MDA, malondialdehyde; SOD, superoxidedismutase; POD, peroxidase; CAT, catalase; APX, ascorbate peroxidase. [file peerj-08-9780-s002.docx]

Table S2 Summary of UNIANOVA (general linear model, univariate) results (*F*-values and significance levels) for the effects of host and growth phase on physiological performance of *M. savatieri*.

|  | df | chl a | chl b | car | TSS | SP | P | CMP | MDA | SOD | POD | CAT | APX |
| --- | --- | --- | --- | --- | --- | --- | --- | --- | --- | --- | --- | --- | --- |
| Host | 1, 12 | 48^**^ | 124^**^ | 5 | 254^**^ | 30^**^ | 3 | 2533^**^ | 65^**^ | 41^**^ | 11^*^ | 96^**^ | 33^**^ |
| Growth phase | 1, 12 | 1691^**^ | 4413^**^ | 140^**^ | 216^**^ | 388^**^ | 172^**^ | 493^**^ | 2 | 0 | 7^*^ | 27^**^ | 10^*^ |
| Host × Growth phase | 1, 12 | 22^**^ | 73^**^ | 5 | 4 | 23^**^ | 0 | 2161^**^ | 38^**^ | 45^**^ | 13^**^ | 24^**^ | 4 |

^*^ The level of significance is *P* < 0.05. ^**^ The level of significance is *P* < 0.01. df, degrees of freedom. chl a, chlorophyll a, chl b, chlorophyll b, car, carotenoid, TSS, total soluble sugar, SP, soluble protein, P, proline, CMP, cell membrane permeability, MDA, malondialdehyde, SOD, superoxide dismutase, POD, peroxidase, CAT, catalase, APX, ascorbate peroxidase.
