# Supplementary material for: The changes of morphological and physiological characteristics in hemiparasitic Monochasma savatieri before and after attachment to the host plant
Source: PeerJ. 2020 Aug 19;8:e9780. doi: 10.7717/peerj.9780 (PMC7443084; doi:10.7717/peerj.9780)
Supplement: Supplemental Information 3 — ** The level of significance is P < 0.01. df, degrees of freedom. [file peerj-08-9780-s003.docx]

Table S3 Summary of UNIANOVA (general linear model, univariate) results (*F*-values and significance levels) for the effects of host and growth phase on root stele diameter and stomatal density of *M. savatieri*.

|  | df | Stele diameter | Stomatal density |
| --- | --- | --- | --- |
| Host | 1, 20 | 342** | 1 |
| Growth phase | 1, 20 | 1709** | 47** |
| Host × Growth phase | 1, 20 | 325** | 0.265 |

^**^ The level of significance is *P* < 0.01. df, degrees of freedom.
