# Supplementary material for: The changes of morphological and physiological characteristics in hemiparasitic Monochasma savatieri before and after attachment to the host plant
Source: PeerJ. 2020 Aug 19;8:e9780. doi: 10.7717/peerj.9780 (PMC7443084; doi:10.7717/peerj.9780)
Supplement: Supplemental Information 4 — Data are presented as the mean ± standard error (n = 6). Different letters for the same indicator indicate statistically significant differences (P < 0.05). Treatments: PE−GJ, M. savatieri growing without a host 8 weeks after sowing; PE+GJ, M. savatieri growing with one G. jasminoides plant 8 weeks after sowing; AE−GJ, M. savatieri growing without a host 16 weeks after sowing; AE+GJ, M. savatieri growing with one G. jasminoides plant 16 weeks after sowing. [file peerj-08-9780-s004.docx]

Table S4 Root stele diameter and stomatal density of *M. savatieri* plants grown with or without a host after 8 and 16 weeks of sowing.

| Treatment | Stele diameter (µm) | Stomatal density (mm^-2^) |
| --- | --- | --- |
| PE-GJ | 49.84±3.54 c | 377.20±11.53 a |
| PE+GJ | 51.95±2.27 c | 365.39±17.74 a |
| AE-GJ | 159.71±6.21 b | 234.42±27.72 b |
| AE+GJ | 331.91±5.72 a | 199.49±28.30 b |

Data are presented as the mean ± standard error (n=6). Different letters for the same indicator indicate statistically significant differences (*P* < 0.05). Treatments: PE-GJ, *M. savatieri* growing without a host 8 weeks after sowing; PE+GJ, *M. savatieri* growing with one *G. jasminoides* plant 8 weeks after sowing; AE-GJ, *M. savatieri* growing without a host 16 weeks after sowing; AE+GJ, *M. savatieri* growing with one *G. jasminoides* plant 16 weeks after sowing.
