# Supplementary figures and images for: The changes of morphological and physiological characteristics in hemiparasitic Monochasma savatieri before and after attachment to the host plant
Source: PeerJ. 2020 Aug 19;8:e9780. doi: 10.7717/peerj.9780 (PMC7443084; doi:10.7717/peerj.9780)

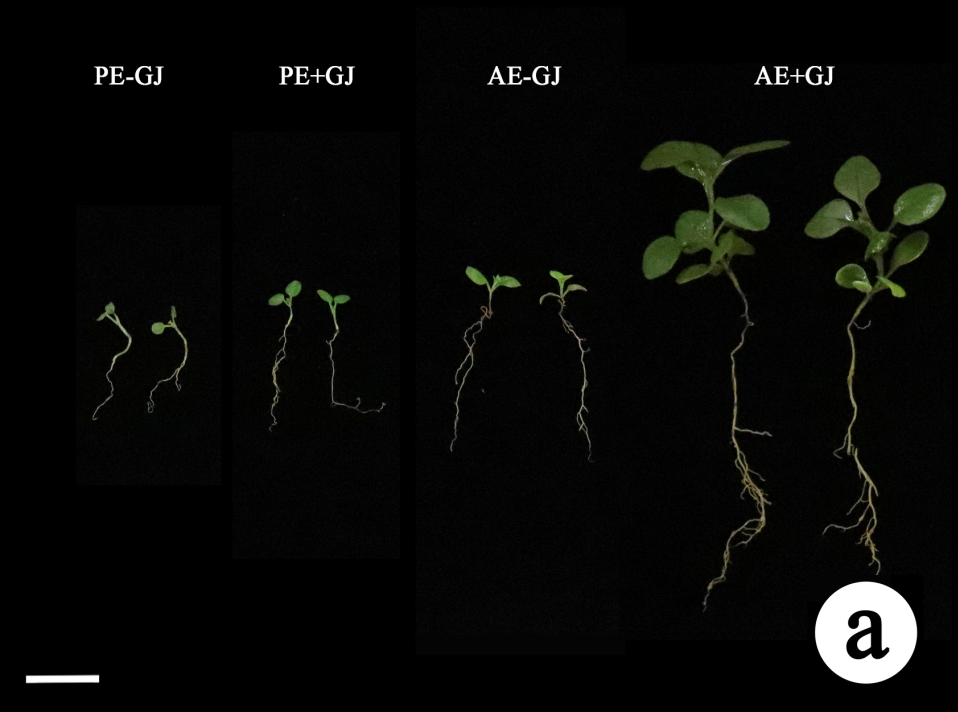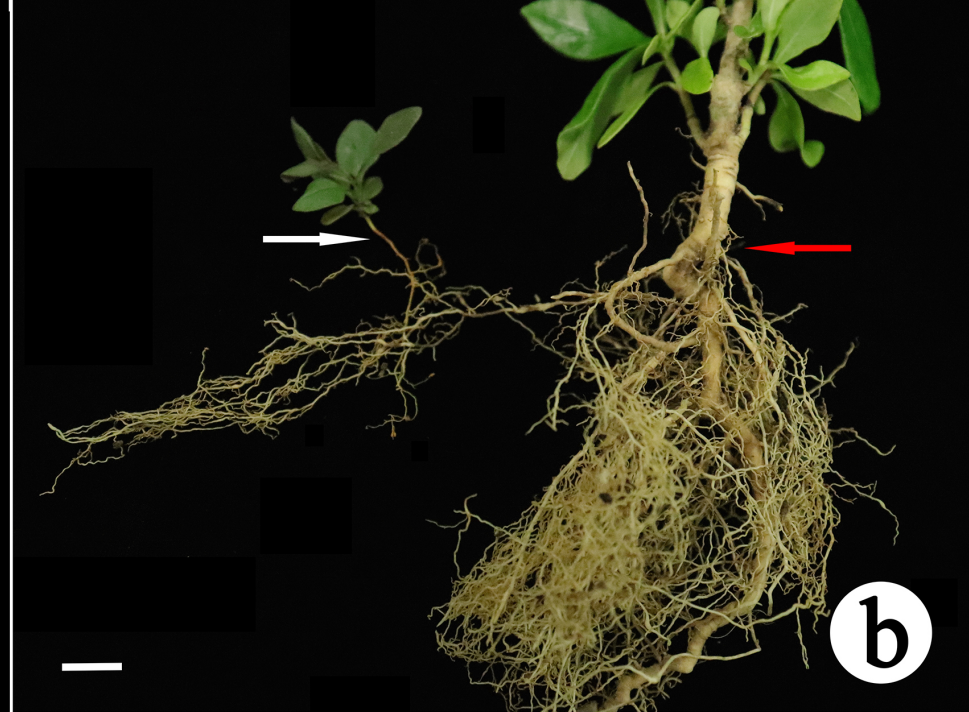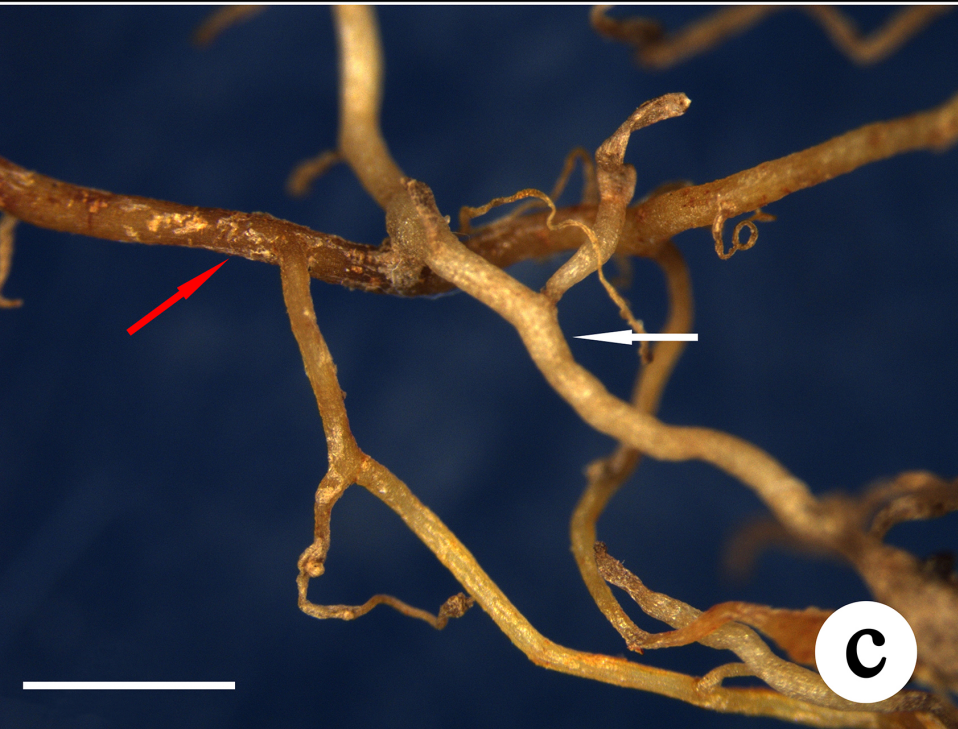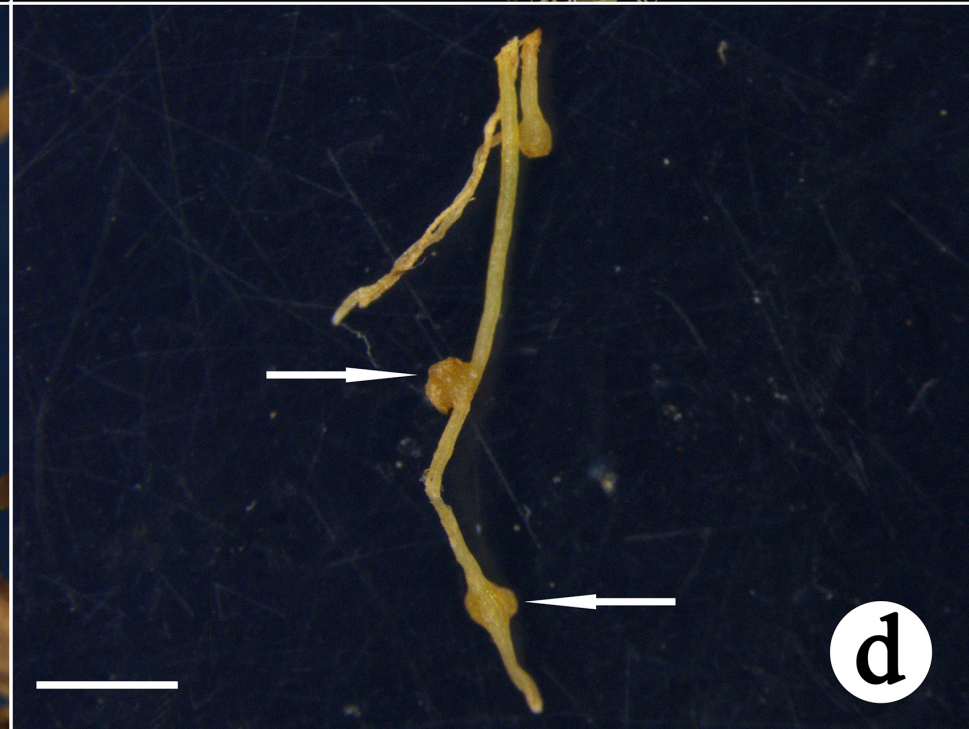

Supplement: Supplemental Information 5 — (A) PE−GJ, M. savatieri growing without a host 8 weeks after sowing; PE+GJ, M. savatieri growing with one G. jasminoides plant 8 weeks after sowing; AE−GJ, M. savatieri growing without a host 16 weeks after sowing; AE+GJ, M. savatieri growing with one G. jasminoides plant 16 weeks after sowing. (B) M. savatieri seedling (white arrow) parasitizing the roots of G. jasminoides (red arrow). (C) Micrograph of the haustorial connection between M. savatieri (white arrow) and G. jasminoides (red arrow). (D) Micrograph of the haustoria of M. savatieri (white arrow) in the absence of a host. (A) and (B) Bar 1 cm. (C) and (D) Bar 1 mm. [file peerj-08-9780-s005.pdf]

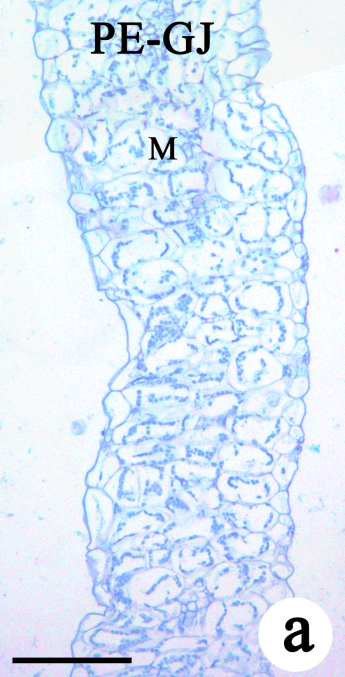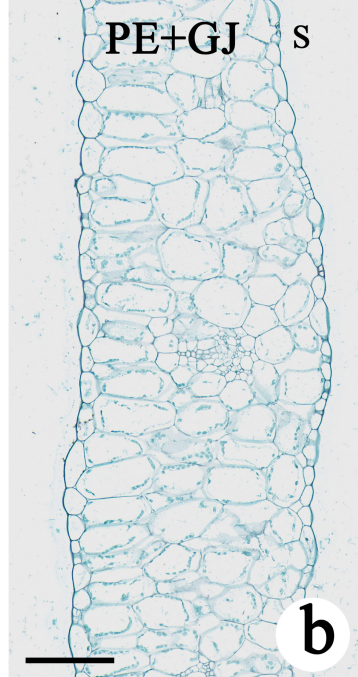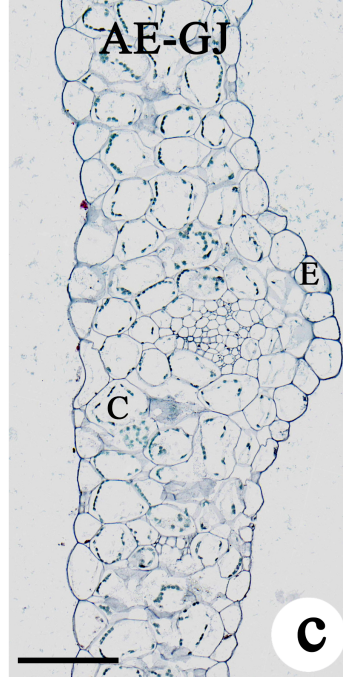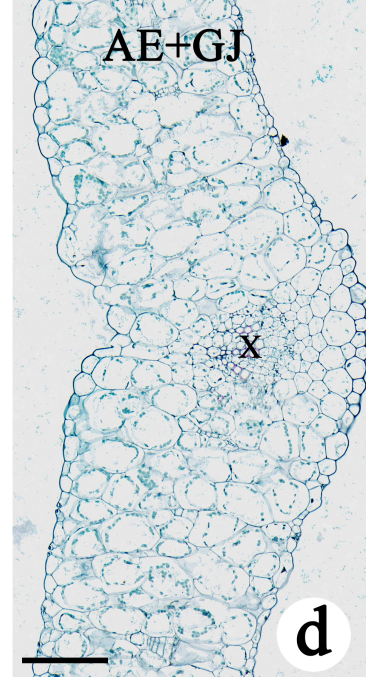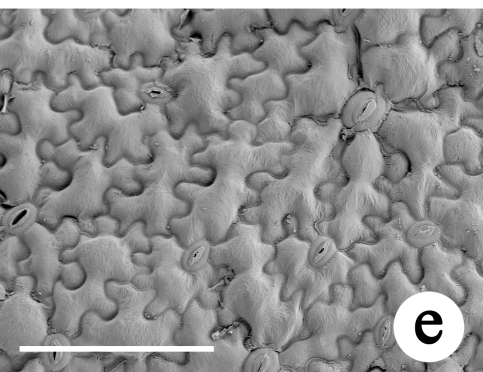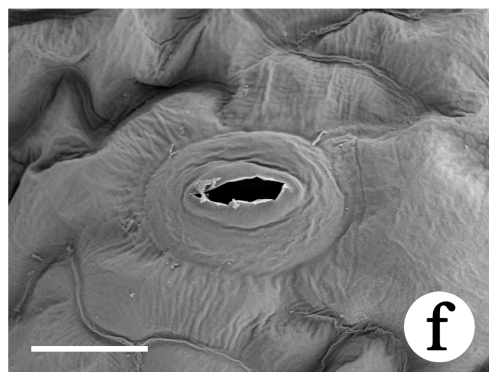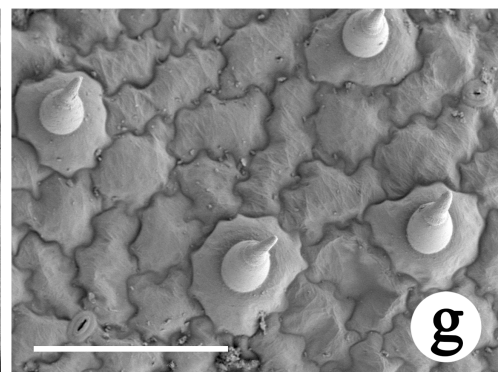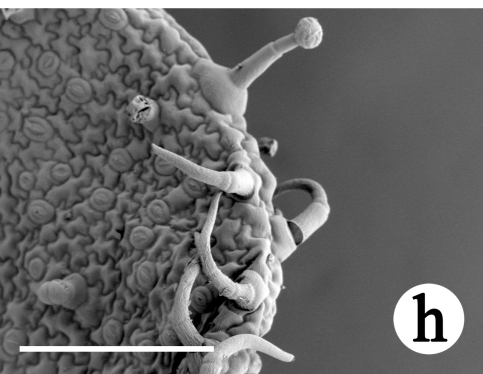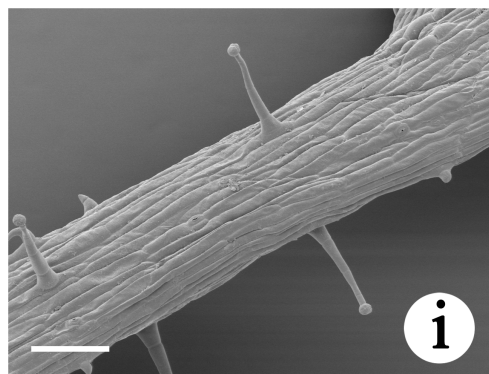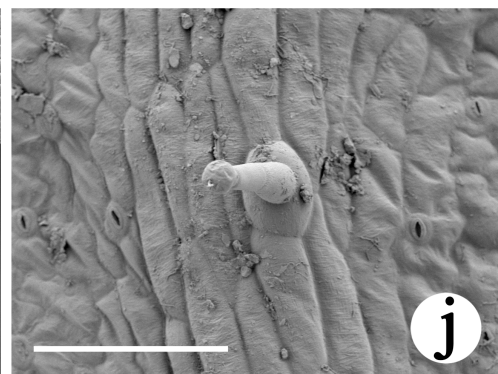

Supplement: Supplemental Information 6 — (A–D) Cross-sections of M. savatieri leaves. Panel a shows M. savatieri growing without a host 8 weeks after sowing. (B) Shows M. savatieri growing with one G. jasminoides plant 8 weeks after sowing. (C) Shows M. savatieri growing without a host 16 weeks after sowing. (D) Shows M. savatieri growing with one G. jasminoides plant 16 weeks after sowing. (E–J) SEM images of M. savatieri leaves. (E) Shows that the epidermis of the leaves was uneven and closely fitted, showing a ridged-like structure. (F) Shows a stomata and two guard cells on the epidermis. (G) Shows that tip-curved papillary epidermal hairs occurred sparsely on the leaves. (H) Shows epidermal hairs and a glandular trichome distributed at the edge of the leaves. (I) Shows epidermal hairs and glandular trichomes distributed on the petioles. (J) Shows glandular trichomes distributed on the leaf vein. C, chloroplast; E, epidermis; M, mesophyll; S, stomata; X, xylem. Bars 100 μm. [file peerj-08-9780-s006.pdf]

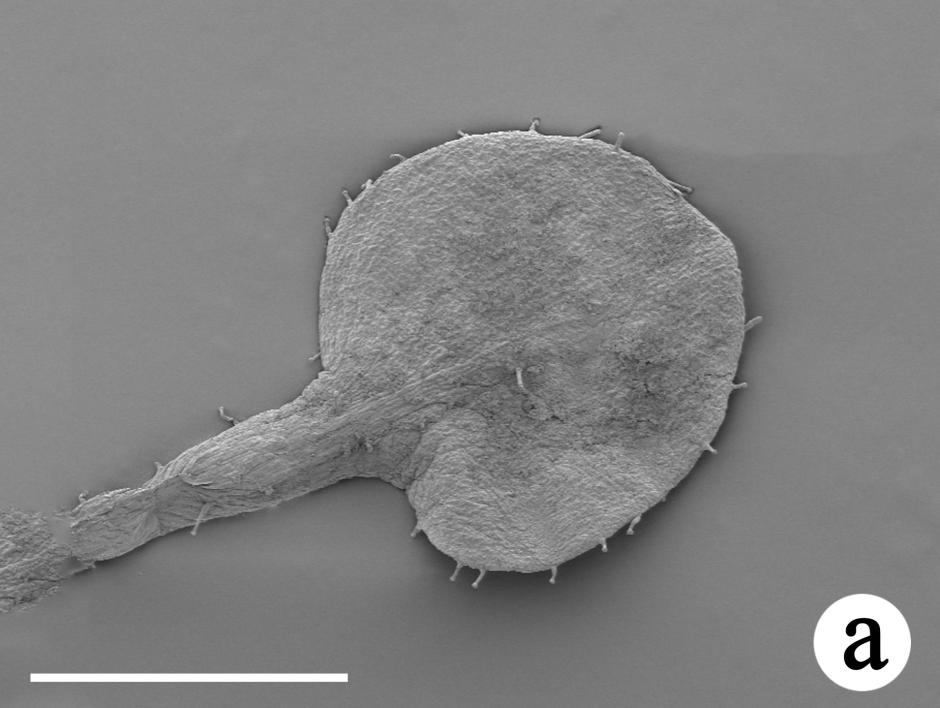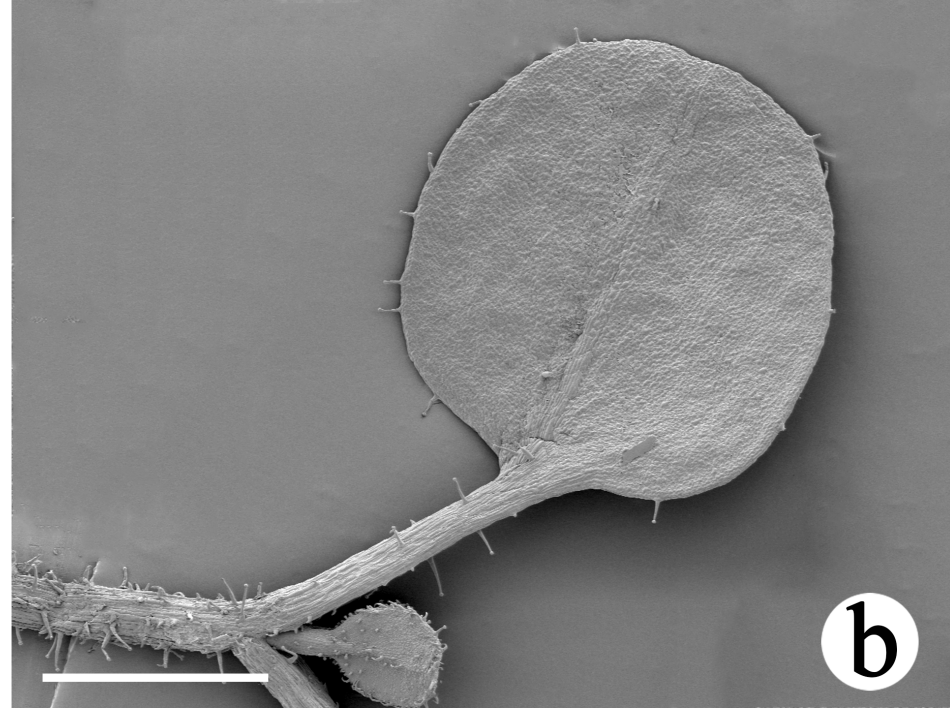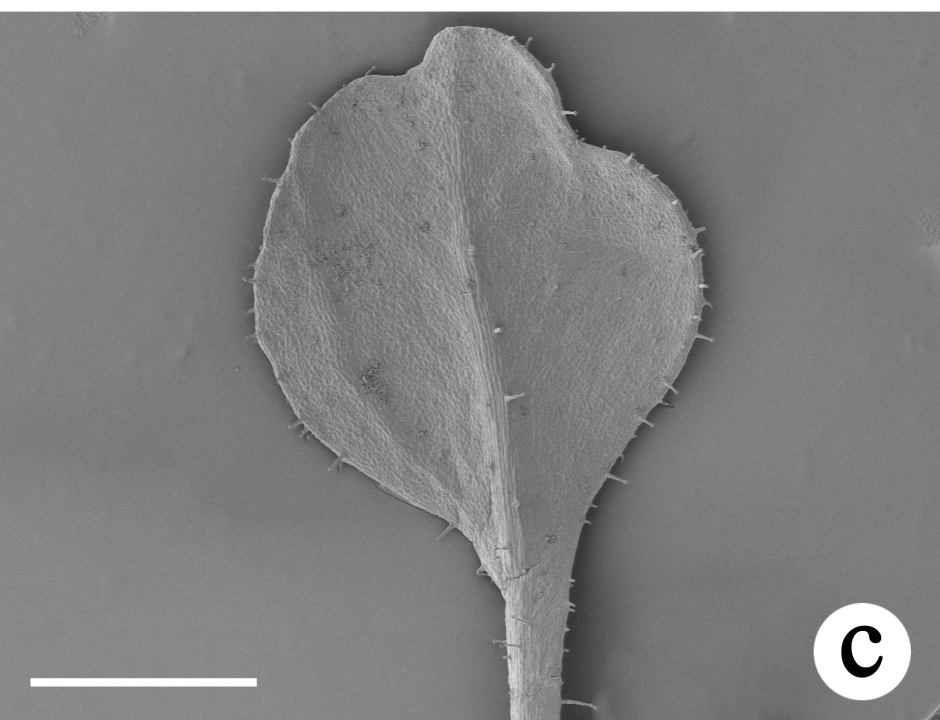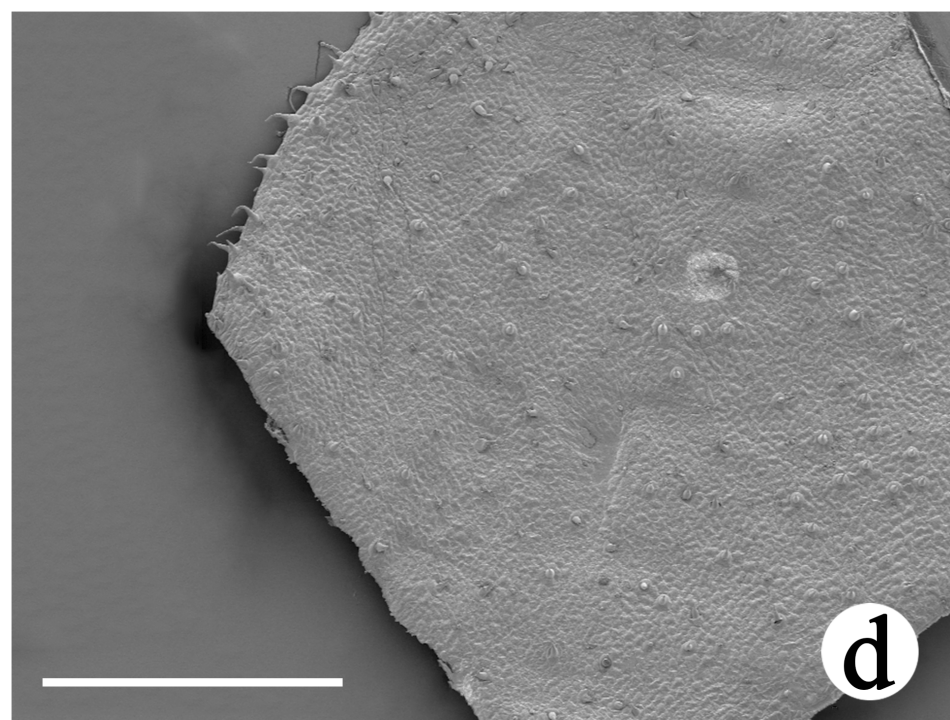

Supplement: Supplemental Information 7 — (A) M. savatieri growing without a host 8 weeks after sowing (PE−GJ). (B) M. savatieri growing with one G. jasminoides plant 8 weeks after sowing (PE+GJ). (C) M. savatieri growing without a host 16 weeks after sowing (AE−GJ). (D) M. savatieri growing with one G. jasminoides plant 16 weeks after sowing (AE+GJ). Bars 1 mm. [file peerj-08-9780-s007.pdf]

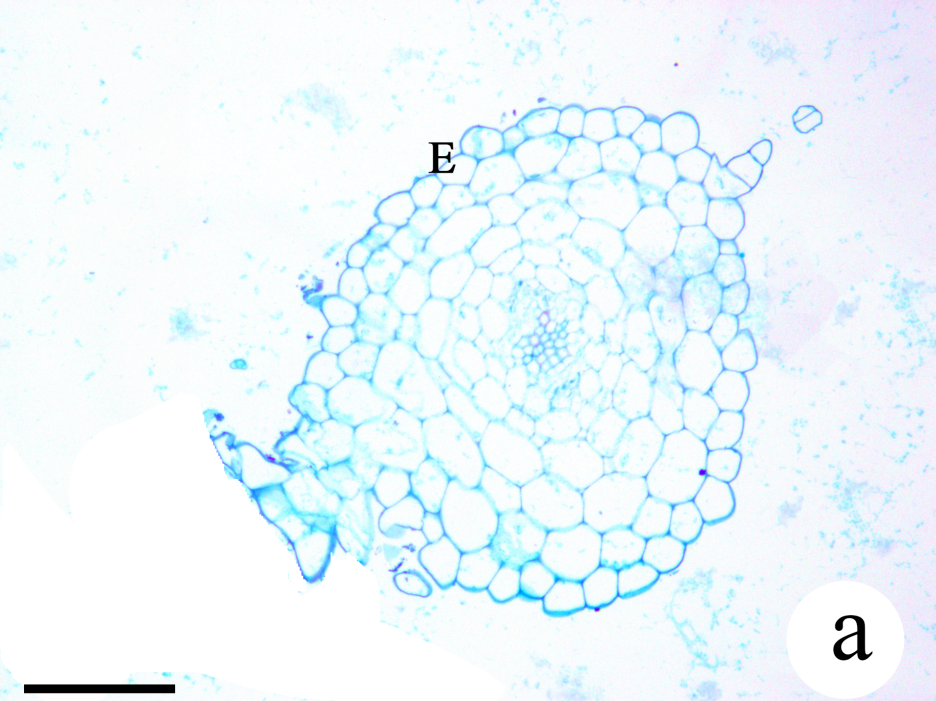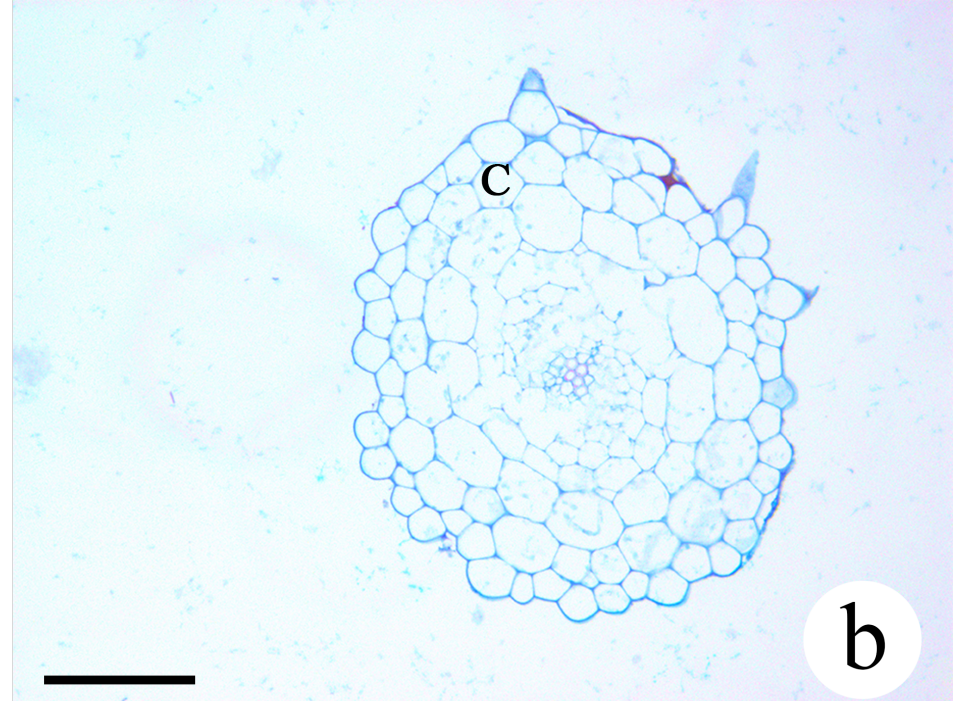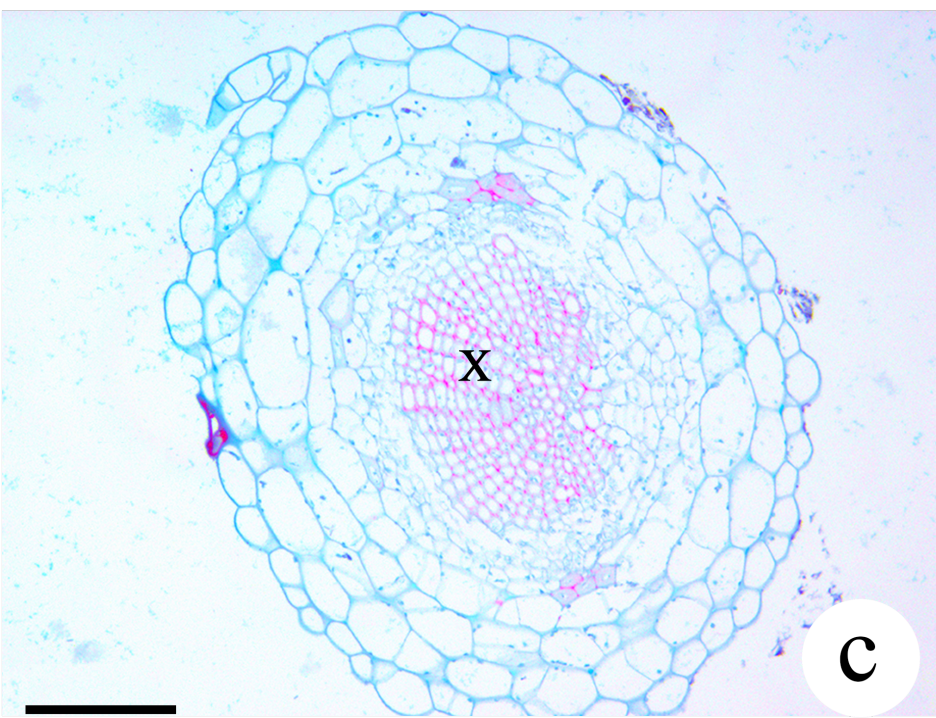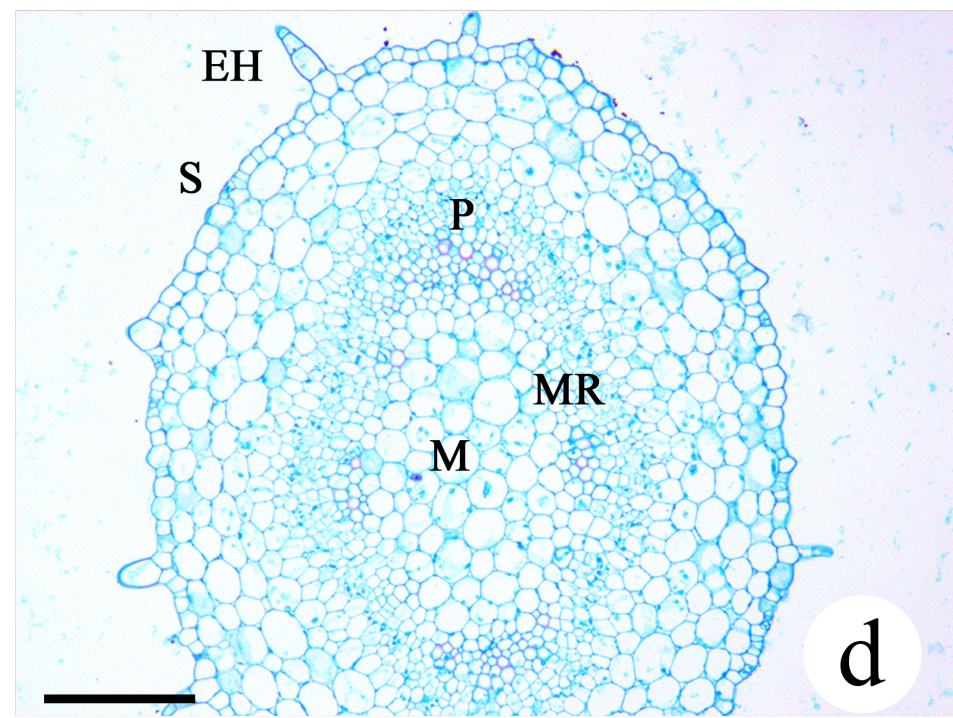

Supplement: Supplemental Information 8 — (A) M. savatieri growing without a host 8 weeks after sowing (PE−GJ). (B) M. savatieri growing with one G. jasminoides plant 8 weeks after sowing (PE+GJ). (C) M. savatieri growing without a host 16 weeks after sowing (AE−GJ). (D) M. savatieri growing with one G. jasminoides plant 16 weeks after sowing (AE+GJ). C, cortex; E, epidermis; EH, epidermal hairs; M, medulla; MR, medullary ray; P, phloem; S, stomata; X, xylem. Bars 100 μm. [file peerj-08-9780-s008.pdf]
